# Management of group b streptococcus-positive pregnant women at maternity homes in JAPAN: a questionnaire survey of compliance among midwives

**DOI:** 10.1186/s40748-017-0069-6

**Published:** 2018-02-05

**Authors:** Kotomi Yamaguchi, Kazutomo Ohashi

**Affiliations:** 1 Department of Human Health Sciences, Kyoto University Graduate School of Medicine, 53 Kawahara-chou, Shougoin, Sakyou-ku, Kyoto, 606-8507 Japan; 2 Present Address: School of Medicine Nursing Course, Gifu University, 1-1 Yanagido, Gifu, 505-1194 Japan; 3 Department of Children and Women’s Health, Division of Health Sciences, Osaka University Graduate School of Medicine, 1-7 Yamada-oka, Suita, Osaka, 565-0871 Japan

**Keywords:** Group B streptococcus, Maternity home, Questionnaire survey, Japan, Screening, Guideline

## Abstract

**Background:**

Per the 2014 Japanese Midwives Association (JMA) guidelines, midwives were allowed to manage the deliveries for group B streptococcus (GBS)-positive pregnant women in labour at maternity homes without the supervision of a medical doctor if they complied with the guidelines of the Japan Society of Obstetrics and Gynecology (JSOG), wherein midwives working for maternity homes are expected to cooperate with commissioned obstetricians and paediatricians in cooperative medical facilities. We examined the rate of compliance with these JMA and JSOG guidelines regarding the management of GBS-positive pregnant women among midwives at maternity homes in Japan.

**Methods:**

Between October and December 2015, an anonymous questionnaire was distributed to 337 maternity homes registered with the JMA by mail. The questionnaire obtained information regarding the timing of GBS screening, specimen collection, transfer of GBS-positive pregnant women from a maternity home to a hospital, administration of intrapartum antibiotic prophylaxis, and collaboration between midwives and commissioned obstetricians. Data were analysed using descriptive statistics. We used frequency distribution as the statistical test.

**Results:**

Responses were received from 246 (73.0%) maternity homes, of which complete responses from 204 maternity homes (valid response rate, 60.5%) were analysed. Of these 204 maternity homes, only 97 (47.5%) conducted a GBS screening test during 33–37 weeks of gestation as recommended by the JSOG guidelines. Although midwives alone managed GBS-positive pregnant women in labour at 135 maternity homes (66.2%), intrapartum antibiotic prophylaxis, as recommended by the JSOG guidelines, was conducted in only 111 (54.4%). Moreover, only 37.0% (50/135) and 82.2% (111/135) of maternity homes ensured that GBS-positive pregnant women in labour with an elapse of ≥18 h after PROM and a body temperature of ≥38.0 °C, respectively, were transferred to a hospital by ambulance. Only at 58.3% (119/204) of maternity homes did midwives discuss the management of labour for GBS-positive pregnant women with commissioned obstetricians.

**Conclusions:**

Some midwives working for maternity homes did not follow the JMA and JSOG guidelines of the management of GBS-positive pregnant women. For improving compliance rates, midwives at maternity homes should discuss the management of GBS-positive pregnant women with commissioned doctors more carefully and concretely per the existing guidelines.

## Background

About 20% of pregnant women develop Group B streptococcus (GBS) colonization around their vagina and/or recto-anal region [1]. 64 infants of 118 colonized pregnant women who does not received intrapartum antibiotics at delivery were colonized with GBS, and the rate of vertical transmission of GBS was 52.5% [2]. GBS causes severe infection such as sepsis, pneumonia, and meningitis in infants under 3 months old. Neonatal GBS infection can be divided into early-onset disease (EOD), occurring within 6 days after birth, and late-onset disease (LOD) occurring, 7 days or later after birth. In the absence of prophylactic treatment for GBS-positive pregnant women, 1–2% of newborns develop EOD by vertical transmission [3].

In 1996, the Centers for Disease Control and Prevention (CDC) recommended a universal screening program for GBS among pregnant women as well as intrapartum antibiotic prophylaxis for GBS-positive pregnant women to reduce the incidence of EOD. On the other hand, in the United Kingdom and New Zealand, a universal and routine antenatal GBS screening method was not recommended; instead, a risk-based GBS prevention strategy was employed [4, 5]. In Japan, the guidelines of the Japan Society of Obstetrics and Gynecology (JSOG) were issued in 2008 following CDC recommendations, JSOG recommended to conduct the GBS screening test between 33 and 37 weeks of gestation in all pregnant women [6]. In a nationwide surveillance study investigated after the guidelines of JSOG, the mortality rates for EOD improved from 14.8% to 11.8%. [7].

Midwives in Japan can manage normal and spontaneous deliveries without the supervision of an obstetrician; however, they cannot prescribe treatment, such as antibacterial agents, or conduct any tests without directions from the medical doctor. Midwives working at maternity homes can handle normal and spontaneous deliveries alone only in the presence of a contract that mandates the cooperation of obstetricians and paediatricians (hereafter, commissioned obstetricians/paediatricians/medical doctors) from medical institutes. Maternity homes see approximately 10,000 births per year (0.9% of the total number of deliveries in 2014). Before 2014, cases of GBS-positive pregnant women going into labour were considered as requiring special attention, such that midwives working at maternity homes would not manage these deliveries without the supervision of an obstetrician. In 2014, the Japanese Midwives Association [8] issued new guidelines according to which midwives working at maternity homes were allowed to handle the deliveries for GBS-positive pregnant women only if they comply with the guidelines of the JSOG and promote cooperation with commissioned obstetricians and paediatricians. However, whether the midwives actually comply with these guidelines is not known.

### Objectives

The aim of this study was to examine the status of compliance with the current guidelines (issued by the Japanese Midwives Association [JMA] and JSOG) for managing GBS-positive pregnant women among midwives working at maternity homes in Japan.

## Methods

### Study design

A cross-sectional study design was used. Ethics approval and consent to participate.

The Ethical Committee of Kyoto University Medical School approved the study (Approval number R0153). Responses to the questionnaire from the maternity homes were considered informed consent. The anonymity of the participants was preserved.

### Setting

Maternity homes in Japan which deal with delivery.

### Participants

We sent an anonymous self-administered questionnaire and an informed consent form to 337 maternity homes registered on the JMA website by mail between September and December 2015. The questionnaire obtained information regarding the numbers of midwives and births per year at each maternity home, and the status of compliance with guidelines regarding GBS-positive pregnant women in terms of (1) the timing of the GBS screening test, (2) the person in charge of specimen collection, (3) the management of GBS-positive pregnant women in labour including transfer from a maternity home to a hospital and administration of intrapartum antibiotic prophylaxis, and (4) the discussion of these issues between midwives and commissioned obstetricians.

Recommendations for managing pregnant women with a GBS infection per the JSOG guideline.

1. Conduct the GBS screening test between 33 and 37 weeks of gestation.
2. Collect a specimen for GBS culture via a lower vaginal and anal canal swab.
3. Administer prophylactic antibiotic treatment to GBS-positive women who are in labour and scheduled to give birth via vaginal delivery or after premature rupture of membranes during pregnancy.

### Date sources

#### JSOG guidelines

Recommendations for managing pregnant women with a GBS infection per the JMA guideline.

Midwives can manage the delivery for GBS-positive pregnant women at maternity homes without the supervision of the obstetrician only if they comply with the guidelines of the JSOG and cooperate with the commissioned obstetricians to prepare for unforeseeable accidents. Moreover, GBS-positive pregnant women in labour should be taken to a hospital via ambulance in the following cases: elapse of ≥18 h after the premature rupture of membranes (PROM) and maternal body temperature of ≥38.0 °C.

#### Statistical methods

The descriptive statistical analyses were carried out with the IBM SPSS Statistics 21.0. (IBM Japan Ltd., Tokyo, Japan). We used frequency distribution as the statistical test.

## Results

### Participants

We received responses to the questionnaire survey from 246 (73.0%) maternity homes and analysed complete responses from 204 maternity homes (valid response rate, 60.5%). Forty-two responses from 32 maternity homes that do not deal with labour and 10 that did not want to participate in the study were excluded.

Overall, maternity homes employed 1–14 midwives, and the most frequent number of midwives employed was 2, in 38.1% of maternity homes. Moreover, 22.8% of maternity homes employed 1 midwife and 16.3% employed 3 midwives. The number of deliveries in 2014 ranged 0–208, and the most frequent number of deliveries was 15, which was reported for 5.4% of maternity homes. The median number of deliveries was 16, and 20.9% of maternity homes reported managing fewer than 5 deliveries in a year.

### Main results

#### Timing of screening and specimen collection

GBS screening tests were conducted twice in 3 (1.5%) maternity homes, and the timing of the second test as used for statistical analysis. Medical professionals in 97 (47.5%) maternity homes conducted the GBS screening test during 33–37 weeks of gestation as recommended by the JSOG guideline. Medical professionals in charge of specimen collection were as follows: commissioned obstetricians in 194 (95.1%) cases, midwives in 6 (2.9%), obstetricians or midwives in 3 (1.5%), and a clinical laboratory technician in 1 (0.5%) (Table 1).

**Table 1:**
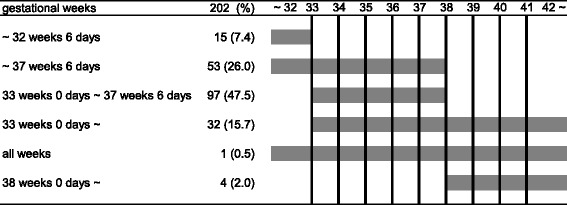
Timing of GBS screening for pregnant women

#### Managing GBS-positive pregnant women in labour

Of the 135 (66.2%) maternity homes wherein midwives handled GBS-positive pregnant women in labour, 111 (82.2%) reported the use of intrapartum antibiotic prophylaxis. Five maternity homes that responded “others” indicated the use of oral antibiotics. Moreover, only 37.0% (50/135) and 82.2% (111/135) of maternity homes ensured that GBS-positive pregnant women in labour with an elapse of ≥18 h after PROM and a body temperature of ≥38.0 °C, respectively, were transferred to a hospital by ambulance (Table 2).

**Table 2:** Management for GBS-positive pregnant women

Manegement	*n* = 135 (%)
Antibiotic prophylaxis during delivery	
Yes	111 (82.2%)
No	16 (11.9%)
Others	6 (4.4%)
No answer	2 (1.5%)
Ambulance transport in case of ≥18 h after the rupture of the membranes	
Yes	50 (37.0%)
No	32 (23.7%)
Others	51 (37.8%)
No answer	2 (1.5%)
Ambulance transport in case of maternal body temperature of ≤38.0 °C	
Yes	111 (82.2%)
No	1 (0.7%)
Others	20 (14.8%)
No answer	3 (2.2%)

#### Collaboration between midwives and commission obstetricians

In only 58.3% (119/204) of maternity homes did the midwives and commissioned obstetricians discuss the management of GBS-positive pregnant women in labour. In 63 (30.9%) maternity homes, the discussion did not take place, and relevant responses were not obtained from 8 (3.9%) maternity homes. Lastly, 9 of 14 maternity homes that provided a response of “others” claimed that discussion for JSOG guidelines regarding the handling of GBS-positive pregnant women in labour at maternity homes was in place before the JMA guidelines were revised in 2014, which midwives could deal with GBG positive pregnant women at maternity homes.

## Discussions

### Key results

In compliance with the JSOG guidelines, only 47.5% of maternity homes reported conducting GBS screening within the recommended period. This percentage was lower than the 64.4% of certified nurse midwives [9] and 97.1% of obstetricians [10] who did comply with the relevant guidelines in the United States. Nevertheless, the recommended timing for screening differs between the JSOG (33–37 weeks of gestation) and CDC guidelines (35–37 weeks of gestation). Although the time window for the test is longer in Japan than in the United States, the compliance rate was lower in midwives working at maternity homes in Japan. In Germany, only 59.3% of pregnant women visiting a tertiary medical care centre underwent a GBS screening test at 35–37 gestational weeks, and the reason for this low compliance rate was reported as the GBS screening test not being covered by medical insurance in Germany [11]. However, the GBS screening test in Japan is covered by the government insurance, which does not explain the low compliance rate observed in Japan.

Additionally, 82.2% of midwives received intrapartum antibiotic prophylaxis, and this percentage was lower than that for certified nurse midwives in the United States (92.5%) as well [9]. In this study, the midwives received prescriptions for oral medicines from the commissioned medical doctors for GBS-positive pregnant women. A safe and efficacious vaccine has not achieved licensure thus far [3, 12, 13], which makes the adequate use of antibiotics essential for the prevention of mother-to-child infection. There is no recommendation regarding the appropriate time for administration of intrapartum antibiotic prophylaxis in the JSOG and JMA guidelines. A previous study conducted in Japan reported that some infants born to GBS-positive women who received antibiotic prophylaxis within 4 h before delivery developed EOD [14], and GBS was detected in these infants [15]. In 2002, the CDC revised the guidelines for intrapartum antibiotic prophylaxis, wherein antibiotics administered at ≥4 h before delivery and any other administrations of antibiotics during pregnancy and labour were deemed unnecessary [3]. However, obstetricians and midwives in the United States were extremely concerned about the timely administration of antibiotics [16]. These results suggest that midwives working at maternity homes in Japan may not be aware of the consequences of GBS infection prophylaxis, find these procedures rather troublesome, or do not ensure adequate collaboration with the commissioned medical doctors. Midwives in Japan cannot conduct any clinical tests or prescribe medicines themselves; therefore, they need to cooperate with commissioned obstetricians in the management of GBS-positive pregnant women.

The CDC provided the algorithm for the secondary prevention of GBS infection in infants and recommended “limited evaluation” including a blood culture at birth, a complete blood count including a differential white blood cell and platelet count at birth and/or 6–12 h after birth, as well as antibiotic administration for infants born to GBS-positive mothers with chorioamnionitis [3]. In cases of inadequate intrapartum antibiotic prophylaxis for GBS-positive pregnant women at ≥18 h after PROM, “limited evaluation” and additional observation for ≥48 h is recommended. The Committee on the Fetus and Newborn also recommended that infants born to GBS-positive mothers ≥18 h after ROM should undergo a blood test [17, 18]. Most cases of EOD demonstrate an acute onset within 24 h, and its prognosis is severe [5, 19]; therefore, early detection and treatment are important. Although the JSOG guidelines do not mention secondary prevention, midwives working at maternity homes should ensure the women’s transfer to a hospital by ambulance when necessary according to the JAM recommendations. In the present study, the rate of compliance with this guideline was very low (37.0%) and some midwives answered that they made arbitrary decisions in cases of 24 h since PROM or consulted a doctor about hospital transfer each time. Moreover, only 82.2% of midwives referred pregnant women in labour to a hospital by ambulance even though fever is an obvious sign of septic infection due to GBS [20, 21]. In such cases, there is a high possibility of a delay in the primary treatment for EOD.

Thus, the compliance with these two guidelines needs to be improved quickly through discussion with commissioned obstetricians. However, only 58.3% of midwives reported discussing the management of GBS-positive pregnant women in labour with commissioned obstetricians. It’s a quite previous study, but it reported that 44% of obstetricians and 39% of neonatologists did not support universal antenatal screening in Australia. [22]. In statement for GBS of Royal Australian and New Zealand College of Obstetricians and Gynaecologists recommended either a risk-based approach or culture-based screening, and midwives at homebirth could not deal with delivery for GBS positive pregnant women or those who not undergo culture-based screening [23]. On the basis of the current findings, we can expect the same situation in Japan. It is important to understand the outcomes of the collaboration between midwives and medical doctors in Japan. Pregnant women have the right to receive adequate treatment and care at any institution, and midwives must therefore comply with the guidelines that recommend cooperation with commissioned doctors. To improve the compliance rate among midwives working at maternity homes, it is also necessary to understand why they do not comply with the recommended guidelines and thus promote their consciousness of risk management.

## Conclusions

In conclusion, more than half of the maternity homes investigated did not conduct the GBS screening test within the gestational period recommended by the JSOG guideline. Despite introducing the new JMA guidelines according to which midwives could manage deliveries for GBS-positive pregnant women at maternity homes themselves only if they complied with the JSOG guideline and cooperate with commissioned doctors, only 58.3% of midwives discussed the management of GBS-positive pregnant women with commissioned obstetricians. Although 66.2% of maternity homes managed GBS-positive pregnant women in labour, only 37.0% ensured transfer of these pregnant women to a hospital in case of ≥18 h had elapsed since PROM. Additional education and promoting discussion with obstetricians for midwives would be essential to improve the current compliance rate.

### Limitations

A limitation of this study should be acknowledged. We included 337 maternity homes opened on the JMA website based on personal information protection out of 441 maternity homes in Japan. Although the response rate was high (73.0%), we conducted this survey within 1 year after the JMA guidelines were revised. Therefore, there is a possibility that midwives and commissioned doctors might not be very familiar with the revised guidelines. Thus, the status of compliance needs to be evaluated continually. Despite this limitation, this study represents the first investigation of compliance with GBS infection-related guidelines among midwives working at maternity homes, and provides useful information in terms of the scope for improvement in the current approach to preventing vertical transmission of GBS.

### Interpretation

The license of obstetrician and midwives are different in each country. Therefore it is necessary for each other to cooperate so that they can conduct evidence-based medical practice in different countries.

## Abbreviations

CDC: Centers for Disease Control and Prevention
EOD: Early-onset disease
GBS: Group B streptococcus
JMA: Japanese Midwives Association
JSOG: Japan Society of Obstetrics and Gynecology
LOD: Late-onset disease
PROM: Premature rupture of membranes
